# Chronic Pain in Italy: Turning Numbers Into Actionable Solutions

**DOI:** 10.1155/prm/3401242

**Published:** 2025-02-14

**Authors:** Alessia Violini, Leonardo Consoletti, Gabriele Finco

**Affiliations:** ^1^Department of Primary Care Director of Palliative Care and Hospice, ASL1 Imperia-Sanremo-Ventimiglia, Liguria, Italy; ^2^Department of Medical and Surgical Sciences, Pain Medicine Unit, University Hospital of Foggia, Foggia, Italy; ^3^Department of Medical Science and Public Health, University of Cagliari, Anesthesia and Intensive Care Therapy, Azienda Ospedaliero Universitaria Cagliari, Cagliari, Italy

**Keywords:** chronic pain, digital health, pain care models, pain center networks, pain medicine specialty, telemedicine

## Abstract

Chronic pain (CP) is a condition that looms over the global social and health scenarios. After many years without having national data, an extensive overview of this disorder in Italy has been published in the first *Rapporto Censis Grünenthal* (Censis Grünenthal Pain Report). It confirms that 19.8% of the Italian adult population suffers from moderate or severe CP, and the vast majority of patients (86.2%) are not aware of the existence of pain management (PM) centers. Starting with the *Rapporto Censis Grünenthal* data analysis by the representatives of three Italian pain scientific societies, several unmet needs were highlighted for which affordable, innovative, and technological strategies were proposed. These solutions focused on interventions in three strategic areas: (1) information, education, and awareness about CP among patients and physicians; (2) organization of pain center networks to adequately cover the national territory, promoting a multimodal-interdisciplinary approach; and (3) use of currently available novel technologies to foster access to treatment. For this purpose, the authors suggested feasible solutions, such as promoting public educational campaigns to raise awareness of the existence of pain centers and the right to receive a proper PM, as indicated in Italy by the pioneering law 38/2010. Regarding organizational gaps, the authors highlighted the possibility of drawing on international models to improve pain centers with completely dedicated staff and community-based pain services while supporting the development of specialized procedural PM standards. Concerning technologies, investments in telehealth and digital tools would improve access to therapies throughout the territory, enabling efficient clinical assessment and helping deliver the most suitable treatments. Overall, greater awareness of the impact of CP and a better allocation of resources are needed to improve patient quality of life, thereby reducing costs for the healthcare system.

## 1. Introduction

CP is the leading cause of disability internationally [1], with a significant emotional burden [2], a decline in quality of life (QoL), a reduction in working abilities resulting in productivity loss and an increase in healthcare costs [3]. The latest estimate of average CP costs in Italy was based on the results from other European countries, as no Italian data were available. It was as high as EUR 4556, of which EUR 1400 was charged to the National Health Service. Productivity loss was estimated at EUR 3157 [4]. The impact of CP in terms of costs is also highlighted in one of the latest published studies on the matter. This study reports that the average annual cost per CP patient in Alberta, Canada, was $5096 (Canadian dollars). Besides this, it showed that 30%–41% of CP patients do not use public healthcare services due to poor accessibility, lack of awareness of available services, or reliance on private services and self-medication [5]. These data underscore the importance of a proper diagnosis and multimodal and multidisciplinary management of CP to avoid deleterious impacts on patients' QoL [6–12].

In Italy, Law 38/2010 recognizes pain management (PM) as a legal obligation and recommends the development of pain therapy centers as well as ensuring the continuity of the diagnostic–therapeutic pathway [13]. However, access to pain care in Italy has proven to be troublesome. Indeed, in 2017, 61.9% of patients were still unaware of their rights to receive pain treatment [14], one in three patients had not received a proper diagnosis of CP, and late referrals to pain therapy centers were very frequent [15]. These data show that poor attention has been paid to CP, which is still not considered a pathology but is relegated to a private sphere [16, 17]. So, despite the high burden of CP, data on the magnitude of the problem were still lacking.

For this reason, a specific survey was carried out by *Censis*, and the results were published in the *Rapporto Censis Grünenthal* (Censis Grünenthal Pain Report) called *“Vivere senza dolore”* (Live without pain), which was released in December 2023 [18]. This report indicates that moderate and severe CP affects approximately 19.7% of the Italian population (9.8 million individuals). CP most commonly affects the lower back and often results from conditions such as osteoarthritis or physical trauma, though nearly 28% of cases have unidentifiable causes. CP significantly diminishes QoL, with over two-thirds of sufferers reporting moderate to severe impacts on daily activities. In addition, besides 50.5% experiencing sleep disturbances, many patients report apathy, emotional instability, anxiety or depression, and strained family and social relationships. Work-related impacts are profound, with many patients reducing hours or even changing jobs. Economically, CP imposes substantial burdens on individuals and society. The annual cost per patient is estimated at €6304 (∼€62 billion in national costs), including healthcare expenses and productivity losses. Despite 70.4% of patients taking medication, 72% report only moderate effectiveness and 37.9% feel their pain is poorly controlled. There is a call among patients for better support: 86.2% are unaware of the existence of pain therapy centers, and 81.7% believe CP should be recognized as standalone pathology. Many feel misunderstood due to the underestimation of CP either by society and healthcare providers. Of note, after a long time without having Italian data available, the *Rapporto ISTISAN 23/28* (ISTISAN Report 23/28), which analyzed a population of 38,775 patients, has also been published [19]. However, the *Rapporto Censis Grünenthal* is peculiar since it focuses on moderate and severe CP and also presents recent estimations of CP social cost [20]. Despite using different scales to assess the pain intensity in the two reports, CP prevalence was very similar [18, 19]. The results of these two reports are in line with international data [5] and with a previous document released by the organization *Cittadinanzattiva* (Active Citizenship) and titled “*Non non siamo nati per soffrire*” (We are not born to suffer). This latter was published in 2020 and showed that 30.5% of Italian patients with CP are not even aware of the existence of pain centers [21]. Data on CP in Italy essentially show the epidemiological stability of the disease and, together with international data, highlight that there are still several unmet needs.

This work critically discusses these newly available data on CP [18], aiming at raising awareness, promoting sensitivity toward CP, and proposing approaches to improve patient management in Italy.

## 2. Methodology

The data from “*Vivere senza dolore*,” *Rapporto Censis Grünenthal* [18] were analyzed and discussed by three experts (thereby also authors of this paper), each belonging to one of the three most representative Italian pain scientific societies with the aim of identifying pain care methods in Italy and finding innovative approaches capable of improving CP treatment.

The analysis focused on the importance of CP and PM education and awareness within society, some feasible organizational strategies, and educational pathways in pain medicine that can provide adequate multidisciplinary PM throughout Italy.

## 3. Results

### 3.1. CP in Italy: Our Opinion on the Adoptable Methods to Improve Patient Care

The recent Italian statistical data on the spread of CP confirm that to date, and 14 years after the enactment of Law 38/2010, very few achievements have been obtained for the real benefit of relieving CP in the population compared with the expectations of social impact. The scenario previously described, in which CP is a widespread, underestimated, and underrated health issue causing a heavy burden and for which patients frequently seek self-medication instead of being managed efficiently by the healthcare system, is essentially unchanged. The Italian scenario seems to have worsened since patients with CP reported feeling increasingly alone [18]. In light of these data, it seems crucial to raise awareness among society, the healthcare system, and all healthcare professionals about CP's burden and to suggest approaches for managing patients. The data of the *Rapporto Censis Grünenthal* that are analyzed and discussed in this work are summarized in Table 1.

### 3.2. The Need to Raise Awareness of CP and Its Management by the Pain Medicine Specialty

Awareness about CP and its management by the PM specialist (PMS) and the existence of pain centers must be increased in the population and among physicians, especially those involved in diagnostic and therapeutic pathways. The Italian scientific societies and associations have embarked on a shared awareness-raising journey highlighting the need to consider the inalienable right of patients with CP not to suffer having access to nononcological CP treatment. This led to the publication of a 10-point document entitled “*Oltre il dolore, manifesto sociale contro la sofferenza*” (beyond pain, a social manifesto against suffering), signed by Italian scientific societies.

The need to invest in research into new pharmacological therapies, to use technological tools to increase the efficiency of the CP care model, and to verify the implementation of Law 38 is also highlighted [22]. Therefore, the authors believe that it would be useful to continue to promote such activities periodically. In addition, the distribution of informational material to be displayed in clinics and sent to families to educate them about the pathways to cope with CP and help with daily activities and to produce institutional advertising-type TV movies on CP could be useful. Also, public or private information campaigns and web pages containing the list of local reference centers would ensure that the patient is adequately informed about the services a pain therapy center can offer. Promoting information campaigns with expert healthcare personnel in secondary schools to prepare future citizens was proposed. The recognition of PM as a specific discipline and the need to activate *Codice* 96 (Code 96) throughout the national territory to establish hospital activities dedicated to PM has been indicated as crucial [23].

In the current Italian scenario, the pathway of patients with CP is suboptimal, as the rate of referral to PMS and pain centers is still low, and patients are managed by different professional figures such as general practitioners (GPs), orthopedists, rheumatologists, physiatrists, and neurologists [17]. Nevertheless, the authors agree with the need for GPs to perform a pain assessment. Also, the timely referral of patients to pain centers instead of other specialties would prevent patients from starting their pathway in pain centers only when they have reached a very severe level of impairment in their daily activities. In pain centers, the PMS, alongside the multidisciplinary team, can provide appropriate pain diagnosis and management [17]. In this context, it must be reiterated that diagnosis delays may have a negative impact on the patient's QoL and may restrict the therapeutic options, negatively affecting the therapy outcomes.

Of note, from the need that emerged from the *Rapporto Censis Grünenthal,* a national toll-free number was established on 22 October 2024 [20]. Through this toll-free number, patients can speak to medical specialists ready to offer advice on how to deal with pain safely and effectively. Also, this service would help direct patients to the nearest pain therapy center best suited to their needs based on the patient's clinical condition and the need for a clinical assessment, minimally invasive therapies, or complex procedures. Therefore, spreading information about this toll-free number is crucial for the best care of CP patients.

The solutions proposed are summarized in Table 2.

### 3.3. Organizational Solutions

Despite the enactment of Law 38 in 2010, an adequate interdisciplinary PM approach that considers the biological and psychosocial components has not been fully implemented. In light of the challenges that CP poses to healthcare systems and societies, national strategies from the United Kingdom [24], the United States of America [25, 26], Australia [27], and Canada [28] have promoted the transformation of healthcare services to provide patients with an efficient care delivery model for CP. To improve CP prevention and management, these countries have started strengthening community-based pain services, developed a series of CP education and training programs for physicians and specialized procedural standards for PM, and have begun to invest in telehealth [27, 28]. In addition to the points just mentioned, which are shared by several countries, the United Kingdom has also adopted a hybrid treatment approach that relies on evidence-based decision-making and considers the preferences of both the healthcare provider and the patient, including the diverse needs of individuals coping with pain [29]. This hybrid approach should be able to comprehensively address the distress and disability experienced by individuals with CP [30]. Significant improvements in patient journeys have been achieved in the United States of America by adopting standardized data entry through web-based reporting and establishing structured clinical teams in which the responsibilities of each member are determined [31]. All these care models provide a patient-centered, evidence- and outcome-driven, multidisciplinary approach to CP assessment and management that focuses on tailored therapies to meet the individual's needs [27, 32–34].

A multimodal, interdisciplinary intervention with the goal of improving function, reducing psychosocial suffering, and enabling return to work should be part of a treatment strategy for patients with CP [7, 35]. Therefore, CP management should combine pharmacological and nonpharmacological approaches and be personalized based on patient characteristics [36, 37].

To provide a specialized, interdisciplinary, patient-centered approach to diagnosing and managing CP by PMS, the authors have highlighted the importance of considering these international strategies as a basis for the creation of new organizational models of care, where it is considered fundamental to guarantee the continuity of treatment and rehabilitation even outside hospitals. In view of this, the creation of structurally independent and adequately trained pain centers with fully dedicated staff can set up an appropriate clinical pathway and promote integration with local healthcare networks, thus guaranteeing therapeutic continuity. In this way, patients would remain linked to the center, and the probability of losing track of them would be significantly lower. These organizational models aim to encourage return to work by adopting the most appropriate CP treatment. For any patient whose pain is not yet under control and who has not regained full working capacity, a temporary working hour reduction proportional to the patient's health state should be considered.

The authors acknowledge that the reorganization of pain therapy centers has so far been poorly incentivized in the Italian context but could be useful in significantly improving the quality of care for citizens suffering from CP. Therefore, the patient must also be directed to settings that best respond to their specific needs, guided by their own diagnostic and therapeutic care pathway [38]. Therefore, establishing centers for CP management where physicians, nurses, therapists, and psychologists collaborate in a multidisciplinary and coordinated manner is essential. These centers not only ensure comprehensive patient care but also facilitate the continuous updating of evidence-based therapeutic protocols through ongoing training of healthcare providers.

The agreement signed by the State-Regions Conference on 27 July 2020 regarding the accreditation of pain therapy networks outlined a model of integration between professional figures and pain therapy centers [39]. Accreditation of pain therapy networks introduces the perspective of strengthening the role of pain centers in the management of CP pain since they are able to provide appropriate services for difficult-to-treat patients and those who need specific expertise and specific treatments. Also, it supports the characterization and standardization of treatment and assistance pathways for the clinical management of complex and advanced chronic conditions. Pain therapy networks could overcome the fragmentation of responses to the multidimensionality of needs, thus providing a more efficient management system [38]. The authors support urging regional coordination to monitor network accreditation criteria and minimum organizational appropriateness standard requirements for pain therapy centers, establish quality indicators, and possibly provide incentives or penalizations/recalls.

The authors draw attention to the current organizational reality of Italian pain centers that need an optimization of their services. Although health and social workers at these centers show excellent patient management skills, organizational difficulties often hinder their activity. Our opinion is that the small number of PMSs makes it necessary to support other professional figures in well-organized entities called “Health Homes,” which are already present on the national territory. Currently, Health Homes include the presence of psychologists, physiotherapists, GPs, and other professionals. This model could enable the interception of patients, favoring the early start of rehabilitation programs.

In addition, it is essential to establish diagnostic and therapeutic care pathways with long-term approaches that include periodic follow-up evaluations shared at the national, regional, and provincial levels, allowing for homogenous therapeutic offers across the territory.

To improve access to care and support its continuity, the use of technological innovations [38], such as telemedicine, which could eliminate the barriers of geographical distance and support personalized treatments, saving time and reducing costs has been suggested. Telemedicine could follow a pathway in which face-to-face visits are interspersed with remote visits [39]. Indeed, it has been shown that telemedicine improved access to care, facilitated the continuity of care, and allowed a better distribution of resources, reducing costs but maintaining the quality of the service [40]. The teleconsultation method has also been proposed, which would allow three or more clinicians to discuss with each other to ensure the best patient management. This care strategy was implemented during and after the COVID-19 pandemic, given the need to reduce face-to-face visits [41].

The authors also discussed the potential of the latest technological innovations in the predictability, monitoring, and management of CP. Such innovative strategies comprise the development of artificial intelligence (AI) algorithms, the use of wearable devices, virtual reality, and digital therapies, such as apps created specifically for the patient that capture real-time data on which the PMS can intervene [38]. Although implemented in some Italian regions, these methods are not yet sufficiently widespread. Furthermore, the availability of video conferencing devices within healthcare services and patient populations is not yet adequate. In Italy, work is underway to create shared digitized medical records, which would be useful data-collecting tools for profiling the patient accessing the pain therapy center based on the type and location of the pain. Moreover, it would allow the collection of epidemiological information and data on the effectiveness of therapies, also enabling the identification of clinical behaviors related to a specific pathology. The authors draw attention to the need to assess and record the characteristics of patients with CP, the effectiveness of treatment, and patient outcomes by creating pain registries, as was carried out with the University Hospital Pain Registry of Oslo [42]. Creating an Italian CP registry is an ongoing project to outline the profile of patients with CP accessing pain centers, detect the percentage of patients who achieve 30%–50% improvement, and identify the profile of patients who improve following treatment. However, the authors call attention to the need for a shared digitized medical record in order to establish the pain registry.

There is also the possibility of using digital therapies, which consist of apps that could be used for educational purposes to manage behavioral habits and enable real-time monitoring of pain intensity, patient compliance, dosages of drug therapy, and outcomes [38]. Also, AI has the potential to revolutionize CP management by improving pain assessment [43], providing recommendations for CP management, guiding the development of effective tailored therapies, and predicting CP progression and responses to therapy. Indeed, AI tools have the potential to recognize pain intensity based on biopotential patterns through devices, such as electrodermal activity sensors, or using physiological variables and functional testing, multimodal neuroimaging, and autonomic metrics. Also, AI tools might predict CP progression through radiographic- or based on MRI-derived radiomics and response to therapies by using neuroimaging markers [44, 45]. The limitations to the applicability of AI tools in the CP field are the limited availability of high-quality data of patients, the complexity of sharing health information, and the cost and difficulties associated with the validation of AI tools in clinical practice [45].

It is worth noting that the National Chronic Disease Plan, created to harmonize the different interventions by sharing a strategic plan with Italian Regions, does not include CP as a chronic disease. This strategic plan identifies uniform approaches to prevention and ongoing assistance, including home care and treatments. In this context, the authors believe that ministerial actions should be undertaken for the recognition of CP as a disability or the possibility of reimbursement of drugs and procedures for the treatment of CP.

The authors have acknowledged that PM is a rather complex pathway that requires more resources. In order to implement this model, resources could be made available through an increase in the *Fondo Sanitario Nazionale* (National Health Fund) with investments from the *Piano Nazionale Ripresa Resilienza* (National Resilience Recovery Plan) and could allow for the recruitment of adequately trained personnel. Thus, an integrated action of referral scientific societies and patient associations could open an institutional dialog to suggest implementing actions to raise awareness of CP and promote investments of more resources in CP treatment. In this way, it would be possible to provide equal and accessible care to all patients with CP. The possibility of a parliamentary question was also raised to verify the measures the government intends to adopt.

The solutions proposed are summarized in Table 3.

### 3.4. Training Courses on Pain Medicine and Patient–Clinician Communication

The discipline of pain medicine is still a neglected area of medical education [46], and there is an urgent need to improve both undergraduate and postgraduate training and make it a compulsory subject [47–49]. The authors underline that to ensure adequate coverage of the national territory, it is essential to guarantee specialized university training. In Italy, to become a PMS, it is necessary to obtain a specialization in anesthesia and resuscitation, intensive care, and pain medicine. However, training in pain medicine is often not adequately mastered. Therefore, the need to optimize specific training at the university level is reported.

The authors believe that a more diverse range of curricula focused on chronic diseases and pharmacological and nonpharmacological therapies for CP would be very useful. Also, they are of the opinion that it may be worth considering adopting specific master's degrees or opening new specialty schools focused on CP management and that education in this field should be continuous. According to the authors, PMSs should follow well-structured training courses designed by international scientific societies that deal with the dissemination of knowledge on pain, such as IASP and EFIC. These paths should include the exact number of clinical cases, processes, procedures, or treatments necessary to obtain the required level of certification. The training should be conducted in various successive steps over time and should include different operational targets by PMS, who will choose the level of qualification he wants to reach. The training will be carried out by PMSs recognized and validated by the agreement between universities and scientific societies and should be the same in all regions and states of the world [50, 51]. According to this way of proceeding, various degrees of expertise in the treatment of CP should be recognized, as also foreseen by the EFIC:

• Basic level: The physician, regardless of his specialization, is able to make a correct diagnosis of CP on the basis of the clinical history and clinical signs presented by the patient and is subsequently able to implement a suitable primary pharmacological treatment.
• Advanced level: The PMS has advanced skills in the nonpharmacological and pharmacological treatment of CP and has acquired soft skills that allow him to manage a pain team.
• Very high level of specialization: The PMS is able to implement neuromodulatory and/or neuropathic pain relief techniques, complementary treatments (biofeedback, hypnosis, etc.) or alternative medicine therapies (acupuncture, shiatsu, etc.) [51].

Furthermore, an Italian survey suggests that it is necessary to gain clinical experience with internships/peer-to-peer activities that, within the healthcare system, enable physicians to be prepared to take on increasing responsibilities in the organizational context of pain therapy centers and networks. It appears clear that, alongside the promotion of clinical training on pain medicine and standardized and shared diagnostic–therapeutic pathways, the presence of the most expert PMS should enable proper knowledge of medical, legal, and economic problems. This work provided the first evidence on the skill profile and related training needs of anesthetists/PMSs, thus paving the way for developing a training program at the national level to improve CP care in Italy [52, 53]. However, CP treatment is not only performed by anesthesiologists but also by other medical specialists (orthopedists, rheumatologists, neurologists, etc.). Therefore, it would be useful to provide specific training in CP for all medical specialists, and, utopianly, it would be necessary to establish postgraduate training in the diagnosis and treatment of pain.

The solutions proposed are summarized in Table 4.

## 4. Conclusions

The *Rapporto Censis Grünenthal* [18] showed that 14 years after the enactment of Law 38, CP remains a highly prevalent condition in the Italian adult population and thus deserves continuous attention. Furthermore, the data highlighted the need for improved care and management of patients with CP, partly because of a limited referral of patients to reference centers for pain treatment.

**Figure 1:**
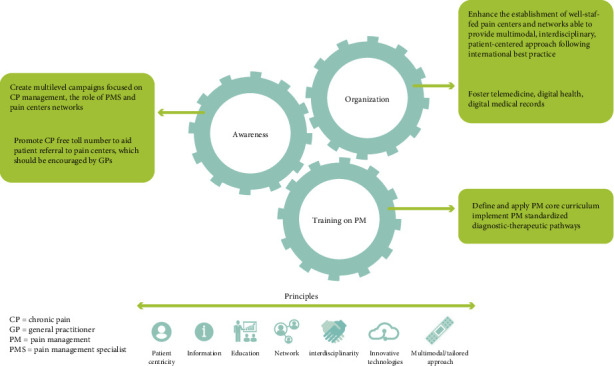
Summary of an integrated plan of feasible solutions to foster a patient-centered high-value CP management. Three areas of intervention were identified, and the main proposed solutions are presented within the green boxes. All areas are interconnected and follow the core principles underneath.

Based on these data, the authors sought feasible solutions that could apply to the current Italian scenario. Thus, the authors highlighted the need for increasing awareness of CP by disseminating information materials, such as brochures and institutional television advertising films, promoting information campaigns, and establishing a national CP day. They also reported that the poor access to pain medicine and pain centers is due to, on the one hand, insufficient coverage of the territory by the pain centers and, on the other hand, the lack of knowledge of which pain centers to contact. Furthermore, the authors believe that to establish the best CP care model, it is essential to adopt a coordinated interdisciplinary and patient-centered approach to ensure individualized management. To implement this strategy, the authors indicated the possibility of following international models of CP care and using digital tools that could help overcome current barriers to optimal treatment. Indeed, the implementation of a CP care model in the United Kingdom, based on a collaborative intervention including a clinical education program, patient assessment, and symptom monitoring, has significantly improved patient outcomes [54]. In the United States of America, the implementation of best-practice patient care processes, including standardization of workflows and the formation of structured clinical teams, has been able to improve the management of CP and facilitate the use of interdisciplinary services [30]. Furthermore, the authors emphasized the need for training courses to ensure sufficient coverage of the territory by pain centers and adequate establishment of pain treatment models.  Figure 1 schematically summarizes the main solutions proposed in this work.

The authors believe that the discussion of the recently published Italian data and the proposals presented in this work, in particular as regards awareness and educational training on CP, will possibly support other countries to help focus on CP with the aim of improving the management of patients. Also, the training model proposed is based on the suggestions of the core curriculum issued by EFIC [51] and could be applicable worldwide.

In conclusion, raising awareness of the impact of CP on patients and the importance of adequate treatment is required. In addition, better resource allocation to establish new pain treatment referral centers and improve the efficiency of the existing ones could enhance patients' QoL while reducing costs.

## Figures and Tables

**Table 1:** Summary of the *Rapporto Censis Grünenthal* data of CP in Italy.

Category	Data
Prevalence of moderate or severe CP in Italy	19.7% (equivalent to 9,800,000 Italians)
Common CP localizations	Lower back: 30.5%; neck: 16.7%; upper back: 15.6%; knee: 13.2%; head: 12.7%; shoulders: 12.6%; legs: 11.6%; feet: 8.1%
Causes of CP	Pathologies: 50.8%; physical trauma: 25.5%; unknown causes: 27.9%
Most common pathology causing CP	Osteoarthritis: 22.3%
Impact on quality of life	67.8% report moderate or severe impact on QoL
Daily activities affected	Lifting objects: 60.2%; physical activities: 59.3%; walking: 49%; household chores: 48.5%; social activities: 36.8%; driving: 23.6%
Additional QoL impacts	Sleep disturbances: 50.5%; relationships with family/friends: 23.2%; sexual relationships: 22.7%; self-care (e.g., dressing): 22.6%; nutrition: 18.6%
Work impact	11.1% unable to work; reduced hours/performance: 40.6%; sick leave: 35.4%; time off: 30.8%; prolonged absences: 27.7%; reduced career opportunities: 25%; job change due to pain: 3.8%; job termination: 1.2%
Treatment effectiveness	70.4% on pain medication; 72% report moderate efficacy; 37.9% report pain not under control; 29.6% not taking medication
Economic burden of CP	Mean annual social cost per patient: EUR 6304 (EUR 1838 direct costs; EUR 4466 indirect costs); total estimated cost of CP in Italy: EUR 62 billion
Patient perspectives on CP	72.5% believe pain is underestimated in society; 36.4% feel their doctor pays little attention to their condition; 56.4% say no one understands how much they suffer; 46.7% report feeling alone with their pain; 7.2% have never discussed CP with anyone; 41.3% feel their pain is seen as an excuse to miss work; 86.2% believe specialized pain services are important in healthcare; 81.7% think pain should be recognized as a distinct pathology

Abbreviations: CP, chronic pain, QoL: quality of life.

**Table 2:** Summary of the solutions identified to increase awareness of CP.

Identified problem	Proposed solutions
Lack of awareness of CP, PMS, and pain therapy networks	Conduct public and private initiatives, including institutional advertising, TV campaigns, and educational efforts in secondary schools
Limited recognition of CP treatment as a right	Promote the right to nononcological CP treatment, as outlined in the “*Oltre il dolore*” manifesto
Low public knowledge of CP coping strategies	Provide educational materials on managing CP and daily coping strategies in clinics and to families
Lack of centralized info on pain centers	Develop online directories to help patients locate nearby pain therapy centers
Difficulty accessing appropriate pain centers	Promote the toll-free number that directs patients to the closest, most suitable pain center for their needs
Limited PM service access across Italy	Implement “*Codice* 96” nationwide to establish hospital-based PM services
Delayed diagnosis and treatment	Promote a timely diagnosis and early referral to pain centers to improve CP care
Low referral rate to PMS and pain centers	Encourage GPs and healthcare providers to conduct pain assessments and refer patients to PMS or pain centers

Abbreviations: CP, chronic pain; GP, general practitioner; PMS, pain management specialist.

**Table 3:** Summary of the organizational solutions identified.

Problem identified	Proposed solution
Lack of an interdisciplinary PM approach	Fully implement a PM approach that integrates biological and psychosocial aspects of CP
Inconsistent national strategies for CP	Adopt international best practices, including community-based pain services, CP education, procedural standards, telehealth, and web-based tools for comprehensive, patient-centered CP management
Inadequate dedicated pain centers	Establish well-staffed pain centers to ensure continuity of CP care and prevent patient dropout
Delayed intervention and rehabilitation	Develop interdisciplinary “health homes” with psychologists, GPs, and specialists to provide early intervention and rehabilitation for CP patients
Inefficient diagnosis and geographical barriers	Expand telemedicine, including teleconsultations, wearable devices, and AI to support pain assessment, personalized CP management and monitoring
Lack of unified medical records for CP patients	Create shared digital medical records and CP registries to track treatment outcomes, collect data, and improve patient profiling
Absence of CP recognition as a chronic disease	Advocate to classify CP as a chronic disease, enabling standardized treatment, home care options, and reimbursement for therapies
Limited resources for CP care expansion	Increase CP funding and staff recruitment through the national health fund and resilience recovery plan; engage government support, including parliamentary advocacy, to prioritize CP treatment
Insufficient government prioritization	Strengthen government focus on CP through parliamentary advocacy and institutional support initiatives

Abbreviations: AI, artificial intelligence; CP, chronic pain; GP, general practitioner.

**Table 4:** Summary of the organizational solutions identified to improve training courses on pain medicine and patient–clinician communication.

Problem identified	Proposed solution
Pain medicine is a neglected area in medical education	- Make pain medicine a compulsory subject in medical training programs (undergraduate and postgraduate)
	- Ensure comprehensive university-level pain management training nationwide
	- Diversify curriculum to include pharmacological and nonpharmacological therapies for chronic pain (CP)
	- Establish master's degrees or specialty programs focused on CP
	- Promote continuous education and lifelong learning for CP professionals
	- Develop targeted training on oncological, musculoskeletal, and lumbar pain, including invasive analgesic techniques
	- Personalize training by proficiency and professional interests
	- Expand clinical experience opportunities (e.g, internships and peer-to-peer activities) in CP care
	- Create a national training program based on identified skill needs in pain medicine
	- Involve expert PMSs for comprehensive CP training across medical, legal, and economic domains
	- Broaden CP training to include more medical specialties to foster cross-disciplinary competence
	- Establish postgraduate training in pain diagnosis and management across specialties

Lack of standardized and shared diagnostic–therapeutic pathways in pain management	Develop and implement standardized diagnostic–therapeutic pathways in pain medicine to ensure consistent patient care

Abbreviations: CP, chronic pain; PMS, pain management specialist.

## Data Availability

Data supporting this study are openly available from Censis site (https://www.censis.it) at the following page https://www.censis.it/sites/default/files/downloads/Vivere%20senza%20dolore_rapporto%20integrale.pdf.
